# Predictors of response to viscosupplementation in patients with hip osteoarthritis: results of a prospective, observational, multicentre, open-label, pilot study

**DOI:** 10.1186/s12891-016-1359-2

**Published:** 2017-01-06

**Authors:** Florent Eymard, Bernard Maillet, Henri Lellouche, Sylvie Mellac-Ducamp, Olivier Brocq, Damien Loeuille, Xavier Chevalier, Thierry Conrozier, on behalf of the Osteoarthritis Group of the French Society of Rheumatology and of the French Research Group in Interventional Rheumatology

**Affiliations:** 1 Department of rheumatology, AP-HP Henri Mondor Hospital, 51 avenue du Maréchal de Lattre de Tassigny, Créteil, 94000 France; 2 Clinique Saint Odilon, Moulins, 03000 France; 3 Department of rheumatology, AP-HP Lariboisière Hospital, Paris, 75010 France; 4 Pole A, Nevers Hospital, Nevers, 58000 France; 5 Department of rheumatology, Princesse Grace Hospital, Monaco, 98000 Monaco; 6 Department of rheumatology and IMoPA (Ingénierie Moléculaire et Physiopathologie Articulaires), Brabois University Hospital, Vandoeuvre-les-Nancy, 54511 France; 7 Department of rheumatology, Nord Franche Comté Hospital, Belfort, 90000 France

**Keywords:** Predictive factors, Hip OA, Viscosupplementation, Joint space narrowing

## Abstract

**Background:**

To identify predictive factors of response to viscosupplementation (VS) in patients with hip osteoarthritis (HOA).

**Methods:**

Prospective, multicentre, open-label trial, achieved in daily practice conditions. Patients with HOA were treated with a single intra-articular injection of a cross-linked hyaluronic acid combined with mannitol (HAnox-M-XL), using imaging guidance. WOMAC pain and function scores and patient global assessment (PGA) were assessed at baseline and day 90. Improvement, satisfaction and efficacy were self-assessed at day 90.

Hip radiographs at baseline were scored using Kellgren-Lawrence grade and Osteoarthritis Research Society International (OARSI) score. Associations between clinical and radiological features and response to VS (pain improvement > 50% at day 90) were assessed in univariate analysis, and then using logistic regression, adjusted for confounding factors.

**Results:**

The intent-to-treat (ITT) population included 97 patients (57 females, mean age 63). Ninety completed the follow-up and 80 had full clinical and radiological data. Response to VS was achieved in 47.8% of patients. In univariate analysis, the only clinical outcome statistically and negatively related to response was PGA at baseline (*p* = 0.047). Radiologically, response to VS was negatively correlated with joint space narrowing (JSN) score (JSN < 2 vs. JSN ≥ 2, *p* = 0.01) and was related to the patterns of femoral head migration (*p* = 0.008). In multivariate analysis, only JSN grade (*p* = 0.03) remained significantly related to a poor response.

**Conclusion:**

This pilot study, which needs further confirmation by larger scale trials, suggests that radiological features might be of importance for the decision of VS in patients with HOA.

**Trial registration number:**

ID RCB N°2013-A00165-40. Registered 31 January 2013.

## Background

Osteoarthritis (OA) is the most common form of articular disease. Its prevalence is increasing and is expected to grow from 11% in 2006 to 25% by 2030 [1], mainly as a consequence of aging population. Hip OA (HOA) is one of the most frequent cause of lower limb OA and is responsible of a significant impact on multiple dimensions of quality of life, compared with healthy controls [2]. HOA was also shown to be associated with an increased risk of all-cause and cardiovascular mortality among older white women [3]. Total hip replacement, which is often the only one solution to alleviate pain in advanced stages of the disease, has been responsible of a dramatic increase of HOA related expenses during the last decade. In patients with mild to moderate HOA, and in those who do not accept or have contra-indication to surgery, pain management includes analgesics, non steroidal anti-inflammatory drugs (NSAIDs), symptomatic slow acting drugs for OA (SYSADOAs), physiotherapy, rehabilitation, intra-articular (IA) steroid injections and viscosupplementation (VS).

VS consists in IA injection (s) of a solution of hyaluronic acid (HA) and aims to alleviate pain and improve joint function, likely by restoring the physiological and rheological homeostasis of OA joints [4, 5]. Recently VS has been suggested to be the most effective treatment for symptomatic knee OA, as attested by an effect-size of 0.63 [6]. Although VS is likely to be effective for the treatment of mild to moderate HOA, literature data provide conflicting results, leading experts to conclude that sufficient evidences are still lacking to recommend VS in the management of symptomatic HOA [7]. However the same authors stressed that a careful analysis of literature shows that most of the negative studies did not respect adequate number of injections (one injection of cross-linked HA or 3 weekly injections of non cross-linked HA) and appropriate indications (mild to moderate radiological OA). On the contrary, the Italian cohort [8], including 1906 patients (4002 injections), strongly suggested a long lasting beneficial effect of ultrasound-guided injections of HA. Based on literature review [9, 10] the major point on which there is overwhelming agreement among the experts is that VS must not be recommended in patients with severe HOA awaiting hip replacement. A retrospective study including 191 patients with HOA, has shown that only 1 out of 4 patients waiting for surgery was satisfied with VS. On the contrary, those who did not consider surgery in the short term had a high success rate (66.6%) [10]. This percentage was similar to that of patients fulfilling the Minimal Clinically Important Improvement (MCII) in an uncontrolled trial, performed in patients with mild to moderate HOA [11]. Anyway prospective randomized controlled trials as well as studies focusing on predictive factors of response according to the OA phenotype remained to be performed [7, 12–16].

The aim of this observational, prospective study, designed by the OA group of the French Society of Rheumatology, was to investigate, in daily clinical practice conditions, the clinical, radiological and technical factors that may, positively or negatively, influence the response to VS in patients suffering from HOA.

## Methods

### Regulatory

The study was carried out in compliance with the principles of Good Clinical Practice (GCP), and the Declaration of Helsinki concerning medical research in humans and the country-specific regulations. Before enrollment, patients were required to sign an informed consent form and were free to withdraw at any time for any reason. The patient informed consent form and the protocol, which complied with the requirements of the International Conference on Harmonisation (ICH), were reviewed and approved by the French *Comité consultatif sur le traitement de l’information en matière de recherche scientifique* (CCTIRS). It was registered 31 January 2013 under the N° ID RCB 2013-A00165-40.

### Study design

PREVICOX was an observational, prospective, multicentre, study, conducted in 25 centers in France, under the aegis of the OA section of the French Society of Rheumatology, between November 2013 and March 2015. Investigators were all rheumatologists belonging to the French Research Group in Interventional Rheumatology (Groupe de Recherche en Rhumatologie Interventionnelle Français, GRRIF). All were highly trained in IA injection techniques.

### Patients

Males and females, fulfilling the American College of Rheumatology criteria for HOA [17] were enrolled if their hip pain failed to respond to analgesics and/or NSAIDs or if they were intolerant to these latter, as recommended by the French Health Autorities. All patients had standard anteroposterior (AP) pelvic X-rays, and Lequesne false profile (LFP) of the target hip [18] and/or MRI examination performed within the previous 6 months (MRI data will be published separately). The main exclusion criteria were a known hypersensitivity to HA or mannitol, a contra-indication for an IA procedure (patients at high risk of hemorrhage or thrombosis, skin or systemic infection) or VS within the prior 3 months and/or IA corticosteroid injection within the previous month in the target hip.

### Treatment

To avoid differences in response rate due to differences of efficacy related to HA formulations, only one viscosupplement was allowed. HAnox-M-XL (marketed as HAppyCross®, LABRHA SAS, Lyon, France) is a viscosupplement, specifically designed for middle-sized joints, that combines a high molecular weight, cross-linked sodium hyaluronate of non-animal origin (16 g/l) with mannitol (35 g/l), supplied in a 2.2 ml syringe. Combination of mannitol to HA might extend the time of contact between HA and the target tissues thanks to its ability to protect HA against ROS-mediated degradation [19]. Only one injection of 2.2 ml was performed for each patient. HANOX-M-XL, kindly provided by the manufacturer, was injected intra-articularly by trained rheumatologists or radiologists under fluoroscopy or ultrasonography guidance, according to the injector preference.

### Data collection

Patients were assessed by the investigator, at baseline (Day 0) and 12 weeks after the date of injection (Day 90). IA HA injection could be carried out during the screening visit or within the next 2 weeks according to the physician and patient schedule.

### Clinical features

At inclusion, demographic and anthropometric data [age, gender, height, weight and body mass index (BMI)] and medical history (disease duration, analgesics and NSAIDs consumption, previous IA injections of HA or corticosteroids) were recorded. Other investigations included the Western Ontario and McMaster Universities Osteoarthritis Index (WOMAC) [20] and patient global assessment (PGA), on a 11 point (0–10) Likert scale, at baseline and D90. At D90, patients self-assessed: 1/level of satisfaction with the treatment (0: not satisfied, 1: little satisfied, 2: satisfied, 3: very satisfied); 2/treatment efficacy (0: no efficacy, 1: mild efficacy, 2: good efficacy; 3: very good efficacy); 3/percentage of improvement (0, <25%, 26–50%, 51–75%, >75%); 4/variation in analgesics/NSAIDs consumption (0, <25%, 26–50%, 51–75%, >75%). At the time of injection, the presence of a synovial fluid effusion was also recorded.

### Radiographic features

Radiographs scoring and morphological evaluation were performed by a single experienced observer (TC). The Kellgren-Lawrence (KL) grade [21] and the OARSI score for joint space narrowing (JSN), osteophytes (Ost), subchondral cysts (SC), femoral head flattening (FHF) were determined with the help of an atlas [22]. For each feature, the selected score was the highest one obtained from the two radiological views (AP or LFP). Kappa value (95% CI) was 0.91 (0.88–0.94) for JSN assessment, 0.79 (0.71–0.87) for Ost, 0.77 (0.67–0.87) and 0.76 (0.68–0.85) for SC [23]. Hips were also classified according to the patterns of femoral head migration: superolateral, superomedial/axial, diffuse, posterior according to Ledingham classification [24]. At last, a morphological evaluation of architectural abnormalities according to the description by Lequesne et al. [25] was made: presence or not of hip dysplasia, femoroacetabular impingement (FAI) and coxa profunda.

### Technical features

The imaging guidance (fluoroscopy or ultrasonography), as well as the kind (complete or relative) and duration (in hours) of the recommended rest, were also recorded. In case of the use of a contrast agent, the amount injected was notified.

### Safety evaluation

Adverse events (AEs) were collected at each visit and categorized using the Medical Dictionary for Regulatory Activities (MedDRA). Definition of adverse events and serious adverse events (SAEs) were in accordance with the European standard EN ISO 14155: 2011. Investigators had to note all AEs on the report form. Any AE had to be assessed by the investigator regarding severity, intensity into “mild, moderate or severe”. In case of SAE, the investigator was required to declare it immediately to the sponsor on a specific form to be sent by fax within 24 h after becoming aware of the event. The investigator also assessed the causal relationship as “excluded” or “not excluded” with the treatment and/or the procedure of IA injection. All AEs whose occurrence could not reasonably be attributed to other causes that the injected treatment, were to be considered as potential reactions to it and the relationship was assessed “not excluded”.

### Response to treatment

Response to treatment was defined in multiple separate ways. For reasons that will be discussed below, “responders” were primarily defined as patients who experienced an improvement >50%, 90 days after injection.

“Responders” were secondarily defined as:

1. Patients who were “very satisfied” or “satisfied” with the treatment.
2. Patients who experienced a decrease in WOMAC pain score > 50%.
3. Patients whose decrease of pain was greater than the MCII [26].
4. Patients whose pain at the end of follow-up was < Patient Acceptable Symptom State (PASS) [27].

### Statistics

XLSTAT® 2015 software (Addinsoft®, Paris, France) was used for statistical analysis.

Baseline and 3-month follow-up characteristics are presented as percentage or median [ranges]. A Mann–Whitney or a chi-square was used to assess the association of quantitative or qualitative factors and response to treatment. Multivariate logistic regression analysis was used to identify independent predictors to VS response and included sex, age, symptoms at baseline and other factors with *P* < 0.20 on univariate analysis. *P* < 0.05 was considered statistically significant.

## Results

### Population

One hundred patients were enrolled in PREVICOX study. The intent-to-treat (ITT) population included 97 patients (3 patients withdrawn from the study before injection) and was constituted of 57 females and 40 males. Ninety patients completed the study (per protocol population). Full clinical and radiographic data were available in 80 patients (X rays population). No significant difference in any demographic and clinical items was found between the 3 populations. Detailed data are summarized in Tables 1 and 2.

**Table 1:** Clinical characteristics of patients with hip osteoarthritis at baseline

	ITT population *N* = 97	PP population *N* = 90	X-Rays population *N* = 80	*P* value
Gender (Females)	58.8%	56.7%	53.2%	>0.05
Age (Years)	63.0 (36.0–87.0)	63.0 (36.0–87.0)	62.0 (36.0–87.0)	>0.05
BMI (kg/m^2^)	25.1 (17.8–41.8)	24.9 (17.8–41.8)	24.9 (17.8–41.8)	>0.05
Disease duration (months)	12.0 (1.0–200.0)	12.0 (1.0–200.0)	12.0 (1.0–200.0)	>0.05
WOMAC pain	26.0 (7.0–42.0)	25.0 (7.0–42.0)	26.0 (7.0–42.0)	>0.05
WOMAC total	120.0 (41.0–194.0)	116.0 (41.0–194.0)	120.0 (41.0–194.0)	>0.05
PGA	7.0 (3.0–10.0)	6.0 (3.0–10.0)	6.0 (3.0–10.0)	>0.05
Previous hip steroid injection	17.5%	16.7%	20.3%	>0.05
Previous hip HA injection	17.5%	17.8%	15.2%	>0.05
NSAIDS regular intake	40.0%	42.2%	38.7%	>0.05
Analgesics regular intake	63.8%	67.0%	64.5%	>0.05

Data are median (range) or percentage of cases. Each WOMAC item was measured on a 11-point Likert scale

*ITT*: intent-to-treat, *PP*: per protocol, *BMI*: body mass index, *WOMAC*: Western Ontario and McMaster Universities Osteoarthritis Index, *PGA*: patient global assessment, *HA*: hyaluronic acid, *NSAID*: non-steroidal anti-inflammatory drug

**Table 2:** Radiographic features in X-Rays population

	X-Rays population
KL grade (*N* = 80)	
I	8
II	33
III	27
IV	12
OARSI grade for JSN (*N* = 80)	
0	4
1	28
2	21
3	27
OARSI grade for Ost (*N* = 79)	
0	4
1	19
2	25
3	31
OARSI grade for SC (*N* = 78)	
0	66
1	12
JSN location (*N* = 79)	
None	4
Superolateral	45
Superomedial-axial	21
Diffuse	3
Posterior	6
Architectural abnormalities (*N* = 77)	
None	42
Dysplasia	6
Femoro-acetabular impingement	15
Coxa profunda	14

Data are number of cases

*KL*: Kellgren-Lawrence, *OARSI*: OsteoArthritis Research Society International, *JSN*: joint space narrowing, *Ost*: osteophytes, *SC*: subchondral cyst

JSN was correlated with disease duration (*p* = 0.009) but not with age, gender, BMI and WOMAC scores. KL score was related to disease duration (*p* = 0.03), WOMAC total score (*p* = 0.001) and PGA (*p* = 0.036) but not with age, gender, BMI and WOMAC pain score. There was a significant association between Ost score and PGA (*p* = 0.02). Total OARSI score (JSN + Ost + SC) was correlated to both PGA and disease duration but not with other items.

### Efficacy outcomes

In ITT population, all clinical outcomes decreased significantly between baseline and month 3. Detailed data are shown in Table 3. At baseline 7% of the patients fulfilled criteria for PASS (26). They were 47% at the end of follow-up. MCII threshold was reached in 50% of cases.

**Table 3:** Clinical assessment at baseline and 90 days after viscosupplementation

	Baseline	Day 90	Baseline vs. Day 90 *P* value
WOMAC Pain	26.0 (7.0–42.0)	16.5 (0.0–46.0)	<0.0001
WOMAC Stiffness	10.0 (0.0–18.0)	6.0 (0.0–17.0)	<0.0001
WOMAC Function	84.0 (23.0–134.0)	58.0 (0.0–133.0)	<0.0001
PGA	7.0 (3.0–10.0)	5.0 (0.0–10.0)	<0.0001

Data are median (range) or percentage of cases. Each WOMAC item was measured on a 11-point Likert scale

PGA, patient global assessment

The decrease of WOMAC pain score was significantly higher in patients with JSN grade 0, 1 and 2 than in those with grade 3 (*p* = 0.003). The same was found when comparing JSN grade 0–1 to grade 2–3 (*p* = 0.01). A similar trend was found for KL score but did not reach statistical significance (KL I-II versus III-IV, *p* = 0.06). The decrease in WOMAC pain score was also correlated with baseline WOMAC pain score (*p* = 0.002), but not with baseline PGA or WOMAC total score (*p* = 0.07 and 0.12 respectively). This outcome was highly correlated with satisfaction and efficacy assessment (*p* < 0.0001).

### Response to treatment

To the question “did you experience any improvement since the time of injection?” 82.2% of patients answered “yes”. Among them, 14.9% reported an improvement of less than 25 and 27.0% reported an improvement between 25 and 50%. Only those who reported an improvement greater than 50% were classified as “responders” (47.8% of the whole population).

Patient’s self-assessment of improvement was highly correlated with the decrease of WOMAC pain and total scores (*p* < 0.00001), level of satisfaction (*p* < 0.00001), patient’s assessment of efficacy (*p* < 0.00001), MCII (*p* < 0.0001) and PASS (*p* < 0.0001). In the PP population 52 patients were satisfied with the treatment and 38 were not. However the number of satisfied patients with the treatment was slightly greater than that of the number of responders according to our definition (57.8% versus 47.8%) showing that the patient’s expectation and feeling are not exclusively and strictly dependent on the symptoms decrease. For instance, it is interesting to mention that 4 out of 7 (57.1%) of patients with the lowest WOMAC pain score at baseline (≤15) were not satisfied, whereas 46 out of 73 (63.0%) with WOMAC pain score between 16 and 35 at baseline were satisfied with the treatment.

### Predictors of response

In univariate analysis, response to treatment was unrelated to gender (*p* = 0.26), BMI (*p* = 0.92) disease duration (*p* = 0.50), NSAIDS or analgesics consumption at baseline (*p* = 0.79 and *p* = 0.49, respectively), previous steroid or HA IA injection (*p* = 0.30 and *p* = 0.19 respectively), imaging guidance (ultrasonography versus fluoroscopy, *p* = 0.60), presence or lack of synovial effusion (*p* = 0.47), recommendation of rest the day following injection (*p* = 0.48), and occurrence of treatment related adverse events (*p* = 0.95). The only clinical data statistically and negatively related to response to treatment was PGA at baseline (*p* = 0.047).

Radiologically, the response to treatment was negatively correlated with JSN score (JSN < 2 mm vs. JSN > 2 mm, *p* = 0.019) and was related to the patterns of femoral head migration (*p* = 0.008). Forty-nine percent of the patients with supero-lateral JSN were classified as responders vs. 63% of those with supero-medial and axial JSN. Only 1 patient out of the 5 with posterior JSN was responder. No correlation was found between response to treatment and Ost score (*p* = 0.74), SC score (*p* = 0.56) and OARSI global score (*p* = 0.28). Correlation between KL score and response did not reach statistical significance (*p* = 0.18). The percentage of responders was 50% in patients without architectural abnormality, 40% in patients with hip dysplasia, 69% in patients with FAI and 62% in patients with coxa profunda. However, these differences did not reach statistical significance (*p* = 0.40).

In multivariate analysis, only JSN grade (*p* = 0.03) remained significantly related to response to treatment, whereas the decrease in WOMAC pain score was correlated to both WOMAC pain score at baseline (*p* = 0.007) and JSN (*p* = 0.01).

### Adverse events

Nine adverse events were reported (9.18%). Three were classified as device/procedure AEs (3.1%). All 3 were described as an increase of the hip pain that occurred the very next hours after injection. Two resolved in less than 24 h with rest and paracetamol. One resolved in 1 week and needed NSAIDs. The other AES were low back pain (2 cases), sciatica (1 case), knee pain in patient with knee OA (1 case) and dizziness (1 case) and were considered as unrelated to the treatment and/or the procedure of injection. One patient, with a very high level of pain at baseline (WOMAC pain = 39/50, PGA = 9/10) underwent total hip arthroplasty during the follow-up.

## Discussion

This pilot study, aimed to identify predictive factors of response of a single IA injection of HAnox-M-XL in patients with hip OA, has chiefly highlighted the role of OA severity as a predictor of efficacy. More than the KL grade, which is a composite index taking into account both JSN and osteophyte, it seems that it is the severity of JSN is the best predictor of response to treatment. In our study, patients with a JSN graded OARSI 2 and 3 had a lower rate of success as compared to patients with grade 0 and 1. This has already been suggested in a previous clinical trial [28].

Moreover, the impact of OA severity on VS failure has been confirmed for the knee joint by the meta-analysis published by Wang et al. [29] and by the recent analysis from the FLEXX database [30]. Moreover, this finding is consistent with the results of a previous study including knee OA patients and showing an inverse relation between VS effectiveness and initial levels of several catabolic biomarkers measured in synovial fluid reflecting OA severity [31]. Literature data do not provide robust data to explain the association between OA severity and VS response. However, previous in vivo and in vitro studies highlighted the anti-inflammatory and pro-anabolic effects of exogenous HA on different joint tissues such as synovium and cartilage, which may contribute to the symptom improvement following VS [32]. However, in case of severe OA lesions, inflammatory and pro-catabolic profile of cartilage and synovium are substantial and so, we may assume that HA properties wont be enough to counteract the deleterious profile of OA tissues. Moreover, in OA joints, the decrease in molecular weight and concentration of HA leads to reduced rheological properties of synovial fluid, which can be partly restore by exogenous HA. But, we can suppose that VS is less efficient when rheologic properties are deeply altered as in severe OA.

Likewise, patients with high WOMAC pain score and PGA score at baseline are likely to have a poorer result than those with medium pain level. Interestingly, patients with low level of pain at baseline had less successful results than those with moderate pain. The most likely hypothesis is that those patients were expecting a complete disappearance of symptoms they did not obtain. Consequently, it seems that patients should have sufficient pain level before treatment in order to perceive improvement as significant.

The other trend that emerges from this study is the role of patterns of femoral head migration. Patients with superomedial and axial JSN are likely to be improved than those with superolateral or posterior JSN. This observation was not confirmed in multivariate analysis probably because of a lack of statistical power related to the too small number of patients. Likewise, despite the lack of statistical significance, the study suggests that the best results are obtained in patients with coxa profunda and FAI, resulting mostly in a superomedial axial JSN. Furthermore patients with hip dysplasia (i.e. with superolateral narrowing) had a lowest rate of success than those with coxa profunda and FAI or without architectural abnormality.

Another interesting point to underline is the absence of influence of the BMI on clinical results. Even in patients with BMI > 30 there was no trend for a poorer efficacy of VS. This confirms that hip and knee OA are two very different entities both in terms of patient profile and in terms of treatments efficacy. Similarly, gender and age are not associated with treatment response and patients satisfaction. More interesting is the lack of influence on response of rest after injection. However the very large majority of physicians advised a relative rest for 24 h and the number of patients who were not advised to have rest is probably too small for showing a statistical difference, if there is one.

The relatively small number of patients is the main limitation of the study. Another one is due to the absence of some radiographic views such as the Dunn view allowing a better evaluation of FAI, particularly for diagnosis of cam-type FAI [33], which may have been underestimated.

## Conclusion

Despite some limitations this prospective study, performed in daily practice conditions, demonstrated that VS with HAnox-M-XL alleviate pain by 50% or more in more than half of patients with hip OA. The best success rate was obtained in patients with mild to moderate JSN, those with moderate pain and disability, and in case of superomedial and axial femoral head migration. This study also suggested that better results are obtained in FAI and coxa profunda than in hip dysplasia. Based on these results it is now possible to design a placebo-controlled trial to determine the real efficacy of hip joint VS.

## Abbreviations

AE: Adverse events
AP: Anteroposterior
BMI: Body Mass Index
CCTIRS: Comité Consultatif sur le Traitement de l’Information en matière de Recherche Scientifique
FAI: Femoroacetabular Impingement
FHF: Femoral Head Flattening
GCP: Good Clinical Practice
GRIFF: Groupe de Recherche en Rhumatologie Interventionnelle Français
HA: Hyaluronic Acid
HAnox-M-XL: Hyaluronic Acid Anti Oxydant-Mannitol-Crosslinked
HOA: Hip Osteoarthritis
IA: Intra-articular
ICH: International Conference on Harmonisation
ITT: Intent to treat
JSN: Joint Space Narrowing
KL: Kellgren-Lawrence
LFP: Lequesne false profile
MCII: Minimal Clinically Important Improvement
MedDRA: Medical Dictionary for Regulatory Activities
MRI: Magnetic Resonance Imaging
NSAID: Non steroidal anti-inflammatory drugs
OA: Osteoarthritis
OARSI: Osteoarthritis Research Society International
Ost: Osteophytes
PASS: Patient Acceptable Symptom State
PGA: Patient Global Assessment
SAE: Serious Adverse Events
SC: Subchondral Cysts
SYSADOA: Symptomatic slow acting drugs for osteoarthritis
VS: Viscosupplementation
WOMAC: Western Ontario and McMaster Universities Osteoarthritis Index
